# A strategy to select suitable physicochemical attributes of amino acids for protein fold recognition

**DOI:** 10.1186/1471-2105-14-233

**Published:** 2013-07-24

**Authors:** Alok Sharma, Kuldip K Paliwal, Abdollah Dehzangi, James Lyons, Seiya Imoto, Satoru Miyano

**Affiliations:** 1 Laboratory of DNA Information Analysis, University of Tokyo, Minato-ku, Tokyo, Japan; 2 School of Engineering, Griffith University, Brisbane, Australia; 3 School of Engineering and Physics, University of the South Pacific, Suva, Fiji

## Abstract

**Background:**

Assigning a protein into one of its folds is a transitional step for discovering three dimensional protein structure, which is a challenging task in bimolecular (biological) science. The present research focuses on: 1) the development of classifiers, and 2) the development of feature extraction techniques based on syntactic and/or physicochemical properties.

**Results:**

Apart from the above two main categories of research, we have shown that the selection of physicochemical attributes of the amino acids is an important step in protein fold recognition and has not been explored adequately. We have presented a multi-dimensional successive feature selection (MD-SFS) approach to systematically select attributes. The proposed method is applied on protein sequence data and an improvement of around 24% in fold recognition has been noted when selecting attributes appropriately.

**Conclusion:**

The MD-SFS has been applied successfully in selecting physicochemical attributes of the amino acids. The selected attributes show improved protein fold recognition performance.

## Background

Discovering the three dimensional structure of a protein from its amino acid sequence via computational means is a challenging task and open for research in biological science and bioinformatics. Deciphering protein structure elucidates protein functions. This has a profound impact on understanding the heterogeneity of proteins, protein-protein interactions and protein-peptide interactions. This further helps in drug design. A usual way to predict the structure of a protein is to first acquire proteins with known structures (e.g. by crystallography techniques) and then from their sequences, the prediction process can be conducted by developing recognition techniques. Thereafter, the developed techniques can be used to classify unknown protein sequences into one of its classes or folds. The length of a protein sequence (i.e., the number of amino acids in it) is usually different from the length of another protein sequence. However, two proteins with different lengths and low sequential similarities can be categorized to the same fold. The identification of protein folds from a protein sequence would bring us one step closer to the recognition of protein structures. A wide range of techniques have been developed over the past two decades to recognize protein folds. Despite numerous contributions and significant enhancements achieved [1,2], the protein fold recognition problem is yet to be completely solved.

The focus in protein fold recognition can be broadly classified into two categories: 1) the development of classifiers to improve fold recognition, and 2) the development of feature extraction techniques using alphabetical sequence (syntactical-based) and/or using physicochemical properties of the amino acids (attribute-based or physicochemical-based). For the former case, several classifiers have been developed or used including linear discriminant analysis [3], Bayesian classifiers [4], Bayesian decision rule [5], K-Nearest Neighbor [6,7], Hidden Markov Model [8,9], Artificial Neural Network [10,11] and ensemble classifiers [1,12]–[14]. For the latter case, several feature extraction techniques have been developed including composition, transition and distribution [15], occurrence [16], pairwise frequencies [17], pseudo-amino acid composition [18], bigrams [19], autocorrelation [6,20,21] and deriving features by considering more physicochemical properties [22].

Dubchak et al. [15] proposed syntactical and physicochemical-based features for protein fold recognition. They used the five following attributes of amino acids for deriving physicochemical-based features namely, hydrophobicity (H), predicted secondary structure based on normalized frequency of α-helix (X), polarity (P), polarizability (Z) and van der Waals volume (V). The features proposed by Dubchak et al. [15] have been widely used in the field of protein fold recognition [4,12,22]–[28]. Apart from the above mentioned 5 attributes used by Dubchak et al. [15], features have also been extracted by incorporating other attributes of the amino acids. Some of the other attributes used are: solvent accessibility [29], flexibility [30], bulkiness [31], first and second order entropy [32], size of the side chain of the amino acids [22]. Several attributes have been picked for feature extraction usually in an arbitrary way for protein fold recognition. Contrary to this, Taguchi and Gromiha [16] argued that features from attributes of amino acids can be ignored due to having insufficient information and only syntactical-based features should be considered. This shows that proper exploration of the amino acid attributes has not been conducted. To this, we posed a question: ‘which of the attributes of the amino acids are to be selected for the protein fold recognition problem?’ The answer to this would open the third category of research apart from 1) the development of classifiers, and 2) the development of feature extraction techniques based on the syntactic and/or physicochemical properties.

In this study, we develop a methodology for selecting the attributes of the amino acids for protein fold recognition in a systematic manner. In order to do this, a successive feature selection (SFS) technique based on an exhaustive greedy search algorithm can be applied [33,34]. The SFS technique can find important features from a group of features. However, since several features could be extracted from an attribute (e.g. composition, transition and distribution from hydrophobicity of amino acids) and there could be many attributes, this would lead to selecting multi-dimensional features belonging to an attribute. Therefore, we develop a scheme to identify important attributes by investigating multi-dimensional features corresponding to attributes. For brevity we call the proposed technique as multi-dimensional SFS (MD-SFS).

We show two schemes of MD-SFS: backward elimination and forward selection. In the backward elimination scheme, the search for the best subset of attributes will start by first retaining all the given attributes. Then an irrelevant attribute is discarded from this subset at an iteration time point that causes minimum loss of information for the subset. This elimination of attributes from a subset is performed until all the attributes are ranked. This scheme is useful to find attributes of low importance that could perform well, if selected in an appropriate subset. In the forward selection scheme, the best attribute is selected first, and a subsequent attribute is included in the subset such that the included attribute improves the performance (e.g., in terms of classification) of the subset. This scheme, however, could be biased towards the highest ranking attribute.

Experiments are carried out using Dubchak’s (DD) dataset [25], Taguchi’s (TG) dataset (Taguchi and Gromiha, [16]) and extended Ding and Dubchak (EDD) dataset [2]. The selection of physicochemical attributes by MD-SFS technique shows improvement in protein fold recognition by around 18 ~ 24% on all the datasets when 10-fold cross-validation has been applied. The MD-SFS technique has been illustrated in the next section and its usefulness has been demonstrated in the subsequent sections.

### Multi-dimensional successive feature selection

**Figure 1:**
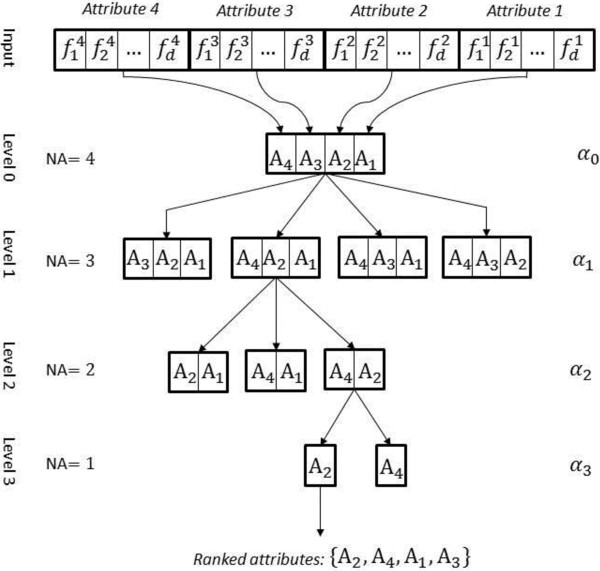
Multi-dimensional successive feature selection: backward elimination scheme.

**Figure 2:**
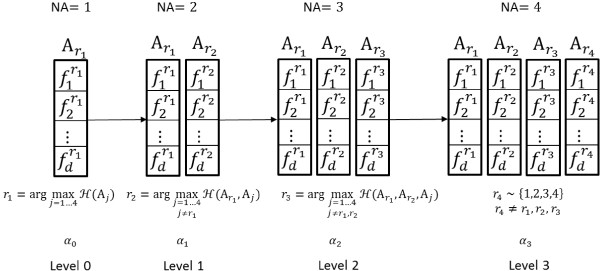
Multi-dimensional successive feature selection: forward selection scheme.

The MD-SFS scheme has been illustrated in Figures 1 and 2. The backward-elimination procedure of MD-SFS has been shown in Figure 1 and the forward-selection procedure has been shown in Figure 2. The purpose of MD-SFS is to select the best attribute for protein fold recognition. In the figures, four attributes (*T*_*a*_ = 4) have been depicted. A feature extraction technique has been used to extract *d*-dimensional features from each attribute. Attributes are represented as A_j_ (where *j* = 1, 2,..., *T*_*a*_) and extracted features of A_j_ are represented as. *f*_1_^*j*^, *f*_2_^*j*^, …, *f*_*d*_^*j*^ In the figures, there are 4 levels in total, including the beginning state. The number of attributes at each of the level is denoted by *NA*. The classification accuracy using *k*-fold cross-validation of a subset of attributes is denoted by *H*( · ) (Figure 2). The highest average classification accuracy using *k*-fold cross-validation at each of the level is depicted by *α*_*l*_ where *l* = 0, 1, …, *T*_*a*_ − 1. The output is the ranked attributes.

### MD-SFS: backward elimination

For the backward-elimination case of MD-SFS (Figure 1), a group of features belonging to an attribute is dropped one at a time in each of the successive levels. This would give subsets of attributes containing features. The number of features in a subset at level *l* is (*T*_*a*_ − *l*)*d*. A classifier is used to compute average classification accuracy using *k*-fold cross-validation procedure on each of the subsets. The subset of attributes with the highest average classification accuracy is progressed to the next subsequent level. The size of subset is reduced by *d* number of features as we progress across the levels. This process is terminated when all the attributes are ranked. In Figure 1, at level 1, the highest average classification accuracy (*α*_1_) obtained is by attribute subset {*A*_1_, *A*_2_, *A*_4_}. It is also possible that average classification accuracy of more than one subset is the same. In that case, the subsets with the highest average classification accuracies would progress to the next level. In Figure 1, subset {*A*_1_, *A*_2_, *A*_4_} is progressed to level 2 and at this level the subset with highest average classification accuracy (*α*_2_) is {*A*_2_, *A*_4_}. At level 3, the subset with highest average classification accuracy (*α*_3_) is {*A*_2_}. In Figure 1, ranked attributes are {*A*_2_, *A*_4_, *A*_1_, *A*_3_}, where A_2_ is the top ranked attribute and A_3_ is the bottom ranked or least important attribute. Furthermore, there could be two criteria in which attributes can be selected. For an instance, if we want to select best 3 attributes for the design then we can take {*A*_2_, *A*_4_, *A*_1_} from the ranked attributes. However, a better way would be to find the argument of the maximum of *α*_*l*_ i.e., $r=\arg\max_{l=0,...,T_{a}-1\;}\alpha_{l}$. For an instance, if r = 2 then this indicates that subset {*A*_2_, *A*_4_} at level 2 exhibits the maximum accuracy among all the selected subsets at all the levels. Therefore, attributes of subset {*A*_2_, *A*_4_} can be selected for the design. We refer the former criterion of selection as brute-n (where *n* is the number of attributes to be selected) and the latter criterion as maximum accuracy (MA) based criterion.

The MD-SFS backward elimination procedure would approximately require between C2Ta+1 and $2^{T_{a}}-1$ search combinations, where *T*_*α*_ is the total number of attributes and the term ^*m*^*C*_*n*_ is the *n*-combination of *m* elements. If *t*_*s*_ denotes the number of attributes in a subset *s* then this subset would have *t*_*s*_*d* features. Therefore, the computational complexity of a classifier for doing classification using subset *s* will be based on *t*_*s*_*d* number of features.

### MD-SFS: forward selection

For the forward-selection case of MD-SFS (Figure 2), an attribute with corresponding *d*-dimensional features would be taken at a time for computing average classification accuracy using the *k*-fold cross-validation procedure. The attribute corresponding to the highest average classification accuracy will be stored; i.e., $r_{1}=\arg\max_{j=1,...T_{a}}H\left(A_{j}\right)$. The selected attribute containing the features will go to the next successive level. In the next level, an attribute that exhibits the highest average classification accuracy in combination with the selected attribute from the previous level $\left(A_{r_{1}}\right)$ will be retained. This process will continue until all the attributes are ranked. The number of features used in computing classification accuracy at level *l* is (*l* + 1)*d*. Further, we can apply the same two criteria (brute-*n* and MA-based) for obtaining attributes from the ranked set of attributes as it was discussed in MD-SFS backward elimination approach.

The MD-SFS forward selection would require around *T*_*a*_(*T*_*a*_ + 1)/2 search combinations, where *T*_*a*_ is the total number of attributes. A subset *s* with *t*_*s*_ attributes would have *t*_*s*_*d* number of features. The computational complexity of a classifier used to compute classification accuracy would depend on *t*_*s*_*d* number of features.

## Methods

### Dataset

In this study, three protein sequence datasets have been used: 1) DD-dataset [25], 2) TG-dataset (Taguchi and Gromiha, [16]) and 3) EDD-dataset [2]. The DD-dataset that we have used consists of 311 protein sequences in the training set where two proteins have no more than 35% of sequence identity for aligned subsequence longer than 80 residues. The test set consists of 383 protein sequences where sequence identity is less than 40%. Both the sets belong to 27 SCOP folds which represented all major structural classes: *α*, *β*, *α*/*β*, and *α* + *β*[25]. The training set and test set have been merged as a single set of data in order to perform *k*-fold cross-validation process.

TG-dataset consists of 1612 protein sequences belonging to 30 different folding types of globular proteins. The names of the number of protein sequences in each of 30 folds have been described in Taguchi and Gromiha [16]. The protein sequences of TG-dataset have been first transformed into their corresponding PSSM (position-specific-scoring-matrix) [35] sequences by using PSIBLAST (http://blast.ncbi.nlm.nih.gov/) (the cut off E-value is set to *E* = 0.001).

EDD-dataset consists of 3418 proteins with less than 40% sequential similarity belonging to the 27 folds that originally used in DD-dataset. We extracted the EDD-dataset from the 1.75 SCOP in similar manner to Dong et al. [2] in order to study our proposed method using a larger number of samples.

### Physicochemical attributes

In this study 30 physicochemical attributes^a^ have been utilized including 5 popular attributes as used by Dubchak et al. [15]. The attributes with the corresponding symbols are listed in Table 1. The residues of amino acids of these 30 attributes are given in Table 2.

**Table 1:** Physicochemical attributes used in the study

**No.**	**Attributes**	**Symbols**
**1**	Hydrophibicity (membrane buried helix) [36]	**H**
**2**	Polarity [37]	**P**
**3**	Polarizability parameter [38]	**Z**
**4**	Normalized frequency of alpha-helix [39]	**X**
**5**	Normalized van der Waals volume [40]	**V**
**6**	alpha-NH chemical shifts [41]	**S**
**7**	A parameter of charge transfer capability [42]	**C**
**8**	The Kerr-constant increments [43]	**K**
**9**	Normalized hydrophobicity scales for beta-proteins [44]	**B**
**10**	Normalized frequency of beta-sheet [45]	**F**
**11**	Normalized frequency of beta-turn [45]	**T**
**12**	Normalized frequency of reverse turn, with weights [46]	**R**
**13**	Size [47]	**E**
**14**	Amino acid composition [48]	**A**
**15**	Frequency of the 1st residue in turn [45]	**F**
**16**	Spin-spin coupling constants 3JHalpha-NH [41]	**N**
**17**	Relative mutability [49]	**M**
**18**	Direction of hydrophobic moment [50]	**D**
**19**	Molecular weight [51]	**W**
**20**	Optical rotation [51]	**O**
**21**	Aperiodic indices for alpha-proteins [52]	**a**
**22**	Aperiodic indices for beta-proteins [52]	**b**
**23**	Aperiodic indices for alpha/beta-proteins [52]	**c**
**24**	Volume [53]	**U**
**25**	Partition energy [54]	**I**
**26**	Heat capacity [55]	**Q**
**27**	Absolute entropy [55]	**L**
**28**	Average accessible surface area [56]	**G**
**29**	Percentage of buried residues [56]	**J**
**30**	Percentage of exposed residues [56]	**Y**

**Table 2:** **Residues of amino acids of the 30 attributes**^**1**^

**No.**	**a**	**c**	**d**	**e**	**f**	**g**	**h**	**i**	**k**	**l**	**m**	**n**	**p**	**q**	**r**	**s**	**t**	**v**	**w**	**y**
1	0.61	1.07	0.46	0.47	2.02	0.07	0.61	2.22	1.15	1.53	1.18	0.06	1.95	0	0.6	0.05	0.05	1.32	2.65	1.88
2	0	1.48	49.7	49.9	0.35	0	51.6	0.13	49.5	0.13	1.43	3.38	1.58	3.53	52	1.67	1.66	0.13	2.1	1.61
3	0.046	0.128	0.105	0.151	0.29	0	0.23	0.186	0.219	0.186	0.221	0.134	0.131	0.18	0.291	0.062	0.108	0.14	0.409	0.298
4	0.486	0.2	0.288	0.538	0.318	0.12	0.4	0.37	0.402	0.42	0.417	0.193	0.208	0.418	0.262	0.2	0.272	0.379	0.462	0.161
5	1	2.43	2.78	3.78	5.89	0	4.66	4	4.77	4	4.43	2.95	2.72	3.95	6.13	1.6	2.6	3	8.08	6.47
6	8.249	8.312	8.41	8.368	8.228	8.391	8.415	8.195	8.408	8.423	8.418	8.747	0	8.411	8.274	8.38	8.236	8.436	8.094	8.183
7	0	0	1	1	0	1	0	0	0	0	0	1	0	0	0	0	0	0	0	0
8	49.1	0	0	0	54.7	64.6	75.7	18.9	0	15.6	6.8	−3.6	43.8	20	133	44.4	31	29.5	70.5	0
9	−0.08	0.76	−0.71	−1.31	1.53	−0.84	0.43	1.39	−0.09	1.24	1.27	−0.7	−0.01	−0.4	−0.09	−0.93	−0.59	1.09	2.25	1.53
10	0.83	1.19	0.54	0.37	1.38	0.75	0.87	1.6	0.74	1.3	1.05	0.89	0.55	1.1	0.93	0.75	1.19	1.7	1.37	1.47
11	0.74	0.96	1.52	0.95	0.66	1.56	0.95	0.47	1.19	0.5	0.6	1.46	1.56	0.96	1.01	1.43	0.98	0.59	0.6	1.14
12	0.77	0.81	1.41	0.99	0.59	1.64	0.68	0.51	0.96	0.58	0.41	1.28	1.91	0.98	0.88	1.32	1.04	0.47	0.76	1.05
13	2.5	3	2.5	5	6.5	0.5	6	5.5	7	5.5	6	5	5.5	6	7.5	3	5	5	7	7
14	8.6	2.9	5.5	6	3.6	8.4	2	4.5	6.6	7.4	1.7	4.3	5.2	3.9	4.9	7	6.1	6.6	1.3	3.4
15	0.06	0.149	0.147	0.056	0.059	0.102	0.14	0.043	0.055	0.061	0.068	0.161	0.102	0.074	0.07	0.12	0.086	0.062	0.077	0.082
16	6.5	7.7	7	7	9.4	5.6	8	7	6.5	6.5	0	7.5	0	6	6.9	6.5	6.9	7	0	6.8
17	100	20	106	102	41	49	66	96	56	40	94	134	56	93	65	120	97	74	18	41
18	0	0.76	−0.98	−0.89	0.92	0	−0.75	0.99	−0.99	0.89	0.94	−0.86	0.22	−1	−0.96	−0.67	0.09	0.84	0.67	−0.93
19	89.09	121.15	133.1	147.13	165.19	75.07	155.16	131.17	146.19	131.2	149.21	132.12	115.13	146.15	174.2	105.09	119.12	117.15	204.24	181.19
20	1.8	−16.5	5.05	12	−34.5	0	−38.5	12.4	14.6	−11	−10	−5.6	−86.2	6.3	12.5	−7.5	−28	5.63	−33.7	−10
21	0.8	0	1.6	0.4	1.2	2	0.96	0.85	0.94	0.8	0.39	1.1	2.1	1.6	0.96	1.3	0.6	0.8	0	1.8
22	1.1	1.05	1.41	1.4	0.6	1.3	0.85	0.67	0.94	0.52	0.69	1.57	1.77	0.81	0.93	1.13	0.88	0.58	0.62	0.41
23	0.93	0.92	1.22	1.05	0.71	1.45	0.96	0.58	0.91	0.59	0.6	1.36	1.67	0.83	1.01	1.25	1.08	0.62	0.68	0.98
24	31	55	54	83	132	3	96	111	119	111	105	56	32.5	85	124	32	61	84	170	136
25	0.1	−1.42	0.78	0.83	−2.12	0.33	−0.5	−1.13	1.4	−1.18	−1.59	0.48	0.73	0.95	1.91	0.52	0.07	−1.27	−0.51	−0.21
26	29.22	50.7	37.09	41.84	48.52	23.71	59.64	45	57.1	48.03	69.32	38.3	36.13	44.02	26.37	32.4	35.2	40.35	56.92	51.73
27	30.88	53.83	40.66	44.98	51.06	24.74	65.99	49.71	63.21	50.62	55.32	41.7	39.21	46.62	68.43	35.65	36.5	42.75	60	51.15
28	27.8	15.5	60.6	68.2	25.5	24.5	50.7	22.8	103	27.6	33.5	60.1	51.5	68.7	94.7	42	45	23.7	34.7	55.2
29	51	74	19	16	58	52	34	66	3	60	52	22	25	16	5	35	30	64	49	24
30	15	5	50	55	10	10	34	13	85	16	20	49	45	56	67	32	32	14	17	41

^1^The first row from column 2 to the last column represents amino acid symbols (‘a,c,d,…,w,y’). The residues correspond to the attributes from Table 1 are given from 2 to the last row.

### Feature extraction

As discussed in the Background Section, there exist several feature extraction techniques. Given a classifier, the features derived from different feature extraction techniques would exhibit different fold recognition performances. Since in this paper the aim is not to find a feature extraction technique for a particular classifier, we use a simple autocorrelation of the residues of protein sequences. The expression for autocorrelation features used in the paper is given as follows:

(1) $$R_{i}=\frac{1}{N}\sum_{k=1}^{N-i}\left(s_{k}-\mu \right)\left(s_{k+i}-\mu \right),$$

where *N* is the length of protein sequence, *s*_*k*_ is the residue of *k*th amino acid in a protein sequence and *μ* is the mean (or average) of *N* residues. In this work, we use *i* = 1, 2, …, 20. Therefore, each protein sequence will give 20-dimensional autocorrelation features.

### Classifiers

In the literature, several classifiers have been used for the protein fold recognition problem. We used three techniques for classification: support vector machine (SVM), Naïve Bayes (NB) and linear discriminant analysis (LDA) with nearest centroid classifier [57]–[59]. SVM and NB classifiers are used from WEKA environment [60] by using WEKA’s default parameter settings.

## Results and discussions

Five attributes used by Ding and Dubchak [25] are used as a benchmark. These attributes are H, P, Z, X and V (see Table 1 for the description of these symbols). In all the experiments we use a 10-fold cross-validation process to obtain the recognition performance. First we present in Table 3 the fold recognition using these 5 attributes on DD, TG and EDD datasets. It can be clearly observed that the highest fold recognition on DD-dataset obtained by HPZXV is 32.8%, on TG-dataset is 28.8% and on EDD-dataset is 38.4%.

**Table 3:** **Protein fold recognition (shown in percentage) on all the datasets using HPZXV attributes used by Ding and Dubchak [**[25]**]**

**DD-dataset**			
**Attribute**	**LDA**	**SVM**	**NB**
HPZXV	23.1%	29.5%	32.8%
**TG-dataset**			
**Attribute**	**LDA**	**SVM**	**NB**
HPZXV	20.5%	23.5%	28.8%
**EDD-dataset**			
**Attribute**	**LDA**	**SVM**	**NB**
HPZXV	27.5%	31.7%	38.4%

Next we apply MD-SFS backward elimination approach on DD-dataset, TG-dataset and EDD-dataset, respectively on three cases: 1) using top 10 attributes of the amino acids from Tables 1, 2) using top 15 attributes of the amino acids from Tables 1, and 3) using all 30 attributes from Table 1. We use two criteria: brute-*n* and MA-based (as discussed in Section MD-SFS: Backward Elimination), to select the attributes. Since in Table 3 the results are reported using 5 attributes, we apply brute-5 to compare the results with that of Table 3. The selected attributes with their corresponding protein fold recognition (abbreviated as PFR in Tables 4, 5, 6, 7, 8, 9, 10 and 11) performance on DD-dataset using brute-5 criterion is given in Table 4 and using MA-based criterion is given in Table 5. The first row of results is by HPZXV (which is taken from Table 3). The first column indicates the number of attributes taken for attribute selection. The same setup has been used for all the remaining tables (Tables 6, 7, 8, 9, 10 and 11). It can be seen from Tables 4 and 5 that incorporating more attributes and then performing attribute selection is helping in improving the recognitionperformance. By using only 5 attributes (Table 4), the recognition performance has significantly improved by 5.7% to 16.6% as compared with the recognition performance of HPZXV attributes. If the number of attributes is not fixed and selection is based on MA criterion then the improvement is recorded between 14.1% and 18.1%.

**Table 4:** MD-SFS backward elimination approach on DD-dataset using brute-5 criterion

**No. of attributes used**	**LDA**		**SVM**		**NB**	
	***Attribute***	***PFR***	***Attribute***	***PFR***	***Attribute***	***PFR***
	HPZXV	23.1%	HPZXV	29.5%	HPZXV	32.8%
1-10	BPCVS	30.2%	BZKSH	31.6%	BZCKP	40.5%
1-15	BZFTP	32.9%	BPKZF	33.3%	BVCKS	38.8%
All	BPEVO	39.7%	BPDFM	35.2%	IUKaP	44.0%

**Table 5:** MD-SFS backward elimination approach on DD-dataset using MA-based criterion

**No. of attributes used**	**LDA**		**SVM**		**NB**	
	***Attribute***	***PFR***	***Attribute***	***PFR***	***Attribute***	***PFR***
	HPZXV	23.1%	HPZXV	29.5%	HPZXV	32.8%
1-10	BPCVS,KXFH	35.0%	BZKSH,XCFV	37.6%	BZCKP,SFH	44.1%
1-15	BZFTP,CEXSK	39.1%	BPKZF,XSCHE,f	40.2%	BVCKS,FPAZR	45.3%
All	LDA-Atr*	39.7%	SVM-Atr*	43.6%	IUKaP, MBbNO	50.9%

*LDA-Atr: BPEVO, XaJRW, AUIQ.

*SVM-Atr: BPDFM, WSHbf, IcXCT, EZaJK, ON.

**Table 6:** MD-SFS backward elimination approach on TG-dataset using brute-5 criterion

**No. of attributes used**	**LDA**		**SVM**		**NB**	
	***Attribute***	***PFR***	***Attribute***	***PFR***	***Attribute***	***PFR***
	HPZXV	20.5%	HPZXV	23.5%	HPZXV	28.8%
1-10	FXBPC	25.4%	FXPVB	29.8%	FXVPH	34.2%
1-15	FBPZV	25.9%	BTAXP	30.4%	BPRfX	37.3%
All	FJBaf	28.3%	JTFQB	31.0%	JbXMK	39.5%

**Table 7:** MD-SFS backward elimination approach on TG-dataset using MA-based criterion

**No. of attributes used**	**LDA**		**SVM**		**NB**	
	***Attribute***	***PFR***	***Attribute***	***PFR***	***Attribute***	***PFR***
	HPZXV	20.5%	HPZXV	23.5%	HPZXV	28.8%
1-10	FXBPC,ZVH	29.8%	FXPVB,CHKS	30.7%	FXVPH,CKSBZ	37.6%
1-15	FBPZV,TCfAE,KSR	32.7%	BTAXP,Zf	33.0%	BPRfX,EKFSA,HCV	41.5%
All	LDA-Atr*	38.6%	SVM-Atr*	36.1%	NB-Atr*	45.3%

*LDA-Atr: FJBaf, ZIEVU, YXPAb, LCQGM, ROW.

*SVM-Atr: JTFQB, AEXCS, IfMLK.

*NB-Atr: JbXMK, aHEPC, YOBVf.

**Table 8:** MD-SFS backward elimination approach on EDD-dataset using brute-5 criterion

**No. of attributes used**	**LDA**		**SVM**		**NB**	
	***Attribute***	***PFR***	***Attribute***	***PFR***	***Attribute***	***PFR***
	HPZXV	27.5%	HPZXV	31.7%	HPZXV	38.4%
1-10	FXPHC	32.6%	BPCZX	36.5%	BXPVF	44.1%
1-15	BTXVZ	33.5%	BTPVC	37.5%	BXPAf	45.7%
All	TJXbV	36.3%	JTFOH	38.2%	IXMEb	46.6%

**Table 9:** MD-SFS backward elimination approach on EDD-dataset using MA-based criterion

**No. of attributes used**	**LDA**		**SVM**		**NB**	
	***Attribute***	***PFR***	***Attribute***	***PFR***	***Attribute***	***PFR***
	HPZXV	27.5%	HPZXV	31.7%	HPZXV	38.4%
1-10	FXPHC,BVSKZ	38.8%	BPCZX,FHKV	39.4%	BXPVF,SKHC	47.7%
1-15	BTXVZ,fFAEP,HCRSK	45.5%	BTPVC,fKXFA,HS	43.3%	BXPAf,KFSZH,CT	51.3%
All	LDA-Atr*	51.8%	SVM-Atr*	47.4%	NB-Atr*	53.9%

*LDA-Atr: TJXbV,GZfUP,BcLHa,YSIQW,DAFEK.

*SVM-Atr: JTFOH,fMCDb,LEaXG,KQBWA,UIS.

*NB-Atr: IXMEb,KGHfC,PABSJ,FWaQc.

**Table 10:** MD-SFS forward selection approach on DD-dataset using brute-5 criterion

**No. of attributes used**	**LDA**		**SVM**		**NB**	
	***Attribute***	***PFR***	***Attribute***	***PFR***	***Attribute***	***PFR***
	HPZXV	23.1%	HPZXV	29.5%	HPZXV	32.8%
1-10	BPCFK	31.9%	BVPKF	32.9%	BKPVC	39.3%
1-15	BPCTV	32.8%	BVPKf	33.1%	BefKC	40.3%
All	BDEFa	35.3%	JBPKG	34.0%	BUDOG	44.1%

**Table 11:** MD-SFS forward selection approach on DD-dataset using MA-based criterion

**No. of attributes used**	**LDA**		**SVM**		**NB**	
	***Attribute***	***PFR***	***Attribute***	***PFR***	***Attribute***	***PFR***
	HPZXV	23.1%	HPZXV	29.5%	HPZXV	32.8%
1-10	BPCFK,ZX	34.7%	BVPKF,HXCZS	37.9%	BKPVC,SFHZ	43.8%
1-15	BPCTV,FKHE	37.4%	BVPKf,XAHTF,SCEZ	39.1%	BEFKC,VPFHT,ASR	44.7%
All	BDEFa,ZPcCQ	40.2%	SVM-Atr*	42.8%	NB-Atr*	50.5%

*SVM-Atr: JBPKG,FUfSC,XEDHa,NTIbZ.

*NB-Atr: BUDOG,baQZI,TMPKN,C.

A similar scheme has been applied using the TG-dataset and the results are reported in Tables 6 and 7 (Table 6 using brute-5 criterion and Table 7 using MA-based criterion). It can be observed from Table 6 that recognition performance has been improved between 7.5% and 10.2%. Also the improvement from Table 7 is between 12.6% and 18.1%.

We have also employed the EDD-dataset for the experiment and the results are reported in Tables 8 and 9 (Table 8 using brute-5 criterion and Table 9 using MA-based criterion). From Table 8, we note that the improvement in recognition performance is between 6.5% and 8.8%, and from Table 9, it is between 15.5% and 24.3%.

Subsequently we applied the MD-SFS forward selection approach on the DD, TG and EDD datasets. Again we use brute-5 and MA-based criteria. The protein fold recognition performance using the DD-dataset with brute-5 criterion is show in Table 10 and with MA-based criterion is shown in Table 11. It can be observed from Table 10 that by using only 5 attributes the recognition performance can be improved between 4.5% and 12.2%. In a similar way, the improvement using MA-based criterion is noted from 13.3% to 17.7%.

On TG-dataset, MD-SFS forward selection with brute-5 criterion is depicted in Table 12 and with MA-based criterion is depicted in Table 13. The improvement from Table 12 using only 5 attributes is between 8.1% and 10.4%; and, from Table 13 we have improvement from 12.4% to 17.5%.

**Table 12:** MD-SFS forward selection approach on TG-dataset using brute-5 criterion

**No. of attributes used**	**LDA**		**SVM**		**NB**	
	***Attribute***	***PFR***	***Attribute***	***PFR***	***Attribute***	***PFR***
	HPZXV	20.5%	HPZXV	23.5%	HPZXV	28.8%
1-10	FXBPC	25.4%	BVFXP	29.9%	BPXVF	34.2%
1-15	FXTBV	26.6%	BTEPX	31.8%	BPEXf	36.6%
All	FJTaB	30.1%	JTFWD	31.6%	JTMWO	39.2%

**Table 13:** MD-SFS forward selection approach on TG-dataset using MA-based criterion

**No. of attributes used**	**LDA**		**SVM**		**NB**	
	***Attribute***	***PFR***	***Attribute***	***PFR***	***Attribute***	***PFR***
	HPZXV	20.5%	HPZXV	23.5%	HPZXV	28.8%
1-10	FXBPC,ZVH	29.8%	BVFXP,CH	30.7%	BPXVF,SKCHZ	37.6%
1-15	FXTBV,ACPZE,fH	33.4%	BTEPX,AfFVS	33.4%	BPEXf,RAKFS,HCV	41.5%
All	LDA-Atr*	38.0%	SVM-Atr*	35.9%	NB-Atr*	45.3%

*LDA-Atr: FJTaB,IUCPG,cLEMO,bHAW.

*SVM-Atr: JTFWD,XBQSI,afcRO,bMAPN,Z.

*NB-Atr: JTMWO,CXBAK,RPUHG,aEQFf,IYb.

Similarly, on EDD-dataset, MD-SFS forward selection with brute-5 criterion is shown in Table 14 and with MA-based criterion is shown in Table 15. The improvement from Table 14 using only 5 attributes is between 7.4% and 8.7%; and, from Table 15 we have improvement from 10.5% to 16.2%.

**Table 14:** MD-SFS forward selection approach on EDD-dataset using brute-5 criterion

**No. of attributes used**	**LDA**		**SVM**		**NB**	
	***Attribute***	***PFR***	***Attribute***	***PFR***	***Attribute***	***PFR***
	HPZXV	27.5%	HPZXV	31.7%	HPZXV	38.4%
1-10	BXCVF	32.5%	BPXFC	36.2%	BXPZF	44.0%
1-15	BTFPE	36.0%	BTPZA	38.0%	BXPAf	45.7%
All	ITXJc	36.2%	ITMJB	39.1%	JTMWF	46.8%

**Table 15:** MD-SFS forward selection approach on EDD-dataset using MA-based criterion

**No. of attributes used**	**LDA**		**SVM**		**NB**	
	***Attribute***	***PFR***	***Attribute***	***PFR***	***Attribute***	***PFR***
	HPZXV	27.5%	HPZXV	31.7%	HPZXV	38.4%
1-10	BXCVF,PHSKZ	29.8%	BPXFC,ZHKV	39.6%	BXPZF,HKSC	47.6%
1-15	BTFPE,CXZAH,fVKRS	33.4%	BTPZA,CXfFK,ERSHV	42.8%	BXPAf,KEFST,H	51.3%
All	LDA-Atr*	38.0%	SVM-Atr*	46.9%	NB-Atr*	54.6%

*LDA-Atr: ITXJc,EGaLB,KbVYC,SDFPO,ZHUfW,AQ.

*SVM-Atr: ITMJB,LOHAa,EXFKC,YDZbR,NfVUG,Q.

*NB-Atr: JTMWF,XKaHP,AOCfD.

From the results, we can deduce that physicochemical based attributes are important for the prediction accuracy of protein folds. An appropriately selected subset of attributes could enhance the prediction accuracy significantly. The subset of attributes selected for different datasets are different. The attributes in a subset also vary depending on the classifier used. However, some attributes repeatedly appear on the obtained subsets. For an instance, a subset BPEVO is selected from all 30 attributes using brute-5 criterion on DD-dataset when LDA is used and a subset BPDFM is selected when SVM is used (see Table 4). It can be observed that the attributes B and P are common in both the subsets. This could imply that these attributes contain more discriminative information for protein fold recognition than others. When we analyzed all the subsets using brute-5 criterion on all the three datasets (Tables 4, 6, 8, 10, 12 and 14), we found that top 5 occurrences of attributes are J (appeared 12 times), B (appeared 9 times), T (appeared 9 times), F (appeared 8 times) and M (appeared 6 times). Therefore, these attributes (J,B,T,F and M) can be seen as important attributes. However, it does not imply that a subset containing all these 5 attributes would perform the best as the performance of attributes in combination with other attributes is also crucial.

We have also carried out a statistical hypothesis test to exhibit the significance of the results achieved. In order to do this, we randomly selected *m* attributes from a given set of *n* attributes and computed prediction accuracy using these *m* attributes. We repeated this random selection *r* times and computed average prediction accuracy. All three classifiers (LDA, SVM and NB) are used for this purpose. We applied this testing on all the three benchmark datasets (DD, TG and EDD) and compared the results with the proposed schemes. In this testing, we used m = 5, n = 30 and r = 20. The results are reported in Tables 16, 17 and 18. It can be observed from these tables that the prediction accuracy using a random selection approach is inferior to the proposed schemes. This depicts that systematically selecting attributes (using MD-SFS procedures) contributed to the prediction accuracy of protein folds.

**Table 16:** Statistical analysis using DD-dataset

**Method**	**LDA**	**SVM**	**NB**
Random selection	9.6%	17.3%	14.7%
MD-SFS forward selection approach	35.3%	34.0%	44.1%
MD-SFS backward elimination approach	39.7%	35.2%	44.0%

**Table 17:** Statistical analysis using TG-dataset

**Method**	**LDA**	**SVM**	**NB**
Random selection	21.2%	25.9%	30.7%
MD-SFS forward selection approach	30.1%	31.6%	39.2%
MD-SFS backward elimination approach	28.3%	31.0%	39.5%

**Table 18:** Statistical analysis using EDD-dataset

**Method**	**LDA**	**SVM**	**NB**
Random selection	29.9%	32.8%	39.8%
MD-SFS forward selection approach	36.2%	39.1%	46.8%
MD-SFS backward elimination approach	36.3%	38.2%	46.6%

Furthermore, we have carried out paired t-test with 5% significance level to study the statistical significance of the prediction accuracy obtained. We used MD-SFS backward elimination method (using brute-5 criterion) as a prototype and used all the three classifiers (LDA, SVM and NB). We compared the results obtained by all the classifiers for HPZXV attributes for DD, TG and EDD benchmarks (the degree of freedom is 2). The paired t-test results for LDA, SVM and NB are 0.029, 0.003 and 0.004, respectively. These results show that the prediction accuracies obtained are significant.

We can summarize that the performance of the protein fold recognition improved when the attributes are appropriately selected. This also shows that physicochemical attributes can play an important role in protein fold recognition if selected appropriately. It should also be noted that the performance can be improved further by considering several other feature extraction techniques with sophisticated ensemble classifiers.

## Conclusion

In this study, we have shown that by selecting physicochemical attributes of amino acids the protein fold recognition performance improved significantly. It is, therefore, beneficial to explore important attributes in the process of determining the three dimensional structure of proteins. To do this, we have developed a multi-dimensional successive feature selection (MD-SFS) technique and shown it on both backward elimination and forward selection approaches. There are several attributes available (e.g. a list of 544 attributes can be found in AAindex, http://www.genome.jp/aaindex/, [61]) and the investigation of these attributes by an exhaustive search would help in solving the problem better. Though it is always useful to explore as many attributes as possible, it comes with an expense of additional computational cost and memory requirements. Nonetheless, computationally efficient techniques for an exhaustive exploration of important attributes should care to develop along with the development of feature extraction and classification techniques.

## Endnote

^a^Though there are large number of physicochemical based attributes defined for amino acids, many authors (e.g. [31,62]–[65]) in the past, used limited number of attributes (up to 8) in their studies. We attempted to study the attributes which were given more emphasis in the literature.

## Competing interests

The authors declare that they have no competing interests.

## Authors’ contributions

AS designed and carried out the experiments, and wrote the first draft of the manuscript. KKP assisted in designing a section of experiments. AD provided the dataset and helped in the second draft of the manuscript. JL also helped in the second draft of the manuscript. SI and SM financed the project. All authors read and approved the final manuscript.
